# Fibroblast Growth Factor Receptor 2: Expression, Roles, and Potential As a Novel Molecular Target for Colorectal Cancer

**DOI:** 10.1155/2012/574768

**Published:** 2012-06-04

**Authors:** Yoko Matsuda, Junji Ueda, Toshiyuki Ishiwata

**Affiliations:** ^1^Departments of Pathology and Integrative Oncological Pathology, Nippon Medical School, 1-1-5 Sendagi, Bunkyo-ku, Tokyo 113-8602, Japan; ^2^Surgery for Organ and Biological Regulation, Graduate School of Medicine, Nippon Medical School, Tokyo 113-8602, Japan

## Abstract

The fibroblast growth factor receptor (FGFR) family consists of four members, named FGFR1, 2, 3, and 4. All 4 FGFRs and their ligands, fibroblast growth factors (FGFs), are expressed in colorectal cancer (CRC). Recent studies have shown that FGFR2 plays important roles in cancer progression; therefore, it is of great interest as a novel target for cancers. Expression of FGFR2 regulates migration, invasion, and growth in CRC. Expression of the FGFR2 isoform FGFR2 IIIb was associated with well-differentiated histological types, and its specific ligand, FGF7, enhanced angiogenesis and adhesion to type-IV collagen via FGFR2 IIIb in CRC. FGFR2 IIIc is detected in CRC, but its roles have not been well elucidated. Interactions between FGFR2 IIIb and IIIc and FGFs may play important roles in CRC via autocrine and/or paracrine signaling. Several kinds of molecular-targeting agents against FGFR2 have been developed; however, it is not clear how a cancer treatment can most effectively inhibit FGFR2 IIIb or FGFR2 IIIc, or both isoforms. The aim of this paper is to summarize the roles of FGFR2 and its isoforms in CRC and clarify whether they are potent therapeutic targets for CRC.

## 1. Introduction

Colorectal cancer (CRC) is the second leading cause of death from cancer in the USA [1] and one of the most serious causes of death from cancer in the world. The prognosis remains poor, especially for patients with advanced or recurrent states of CRC. To improve the survival rates of patients with advanced stages, several types of anticancer, molecular-targeted agents have been developed and are currently undergoing clinical trials [2].

The fibroblast growth factors (FGFs) are heparin-binding growth factors and are classified as FGF-1 to FGF-23 [3–5]. Human FGFs, which comprise ~150–300 amino acids, have a conserved ~120 amino acid residue core, containing ~30–60% amino acid identity [4]. Patients with CRC have been reported to overexpress FGF-1 (acidic FGF), FGF-2 (basic FGF), FGF-3, FGF-7 (keratinocyte growth factor/KGF), FGF-9, FGF-10, FGF-18, FGF-19, and FGF-20. FGFs exert their biological activities by binding to high-affinity tyrosine kinase FGF receptors (FGFRs) on the surface of cells and low-affinity heparan sulfate proteoglycans that enhance ligand presentation [4]. FGFRs consist of four members, named FGFR1, 2, 3, and 4, that are encoded by distinct genes. FGFRs are single transmembrane receptors, containing extracellular, transmembrane, and intracellular domains. The extracellular domain of FGFRs is usually composed of 3 immunoglobulin-like domains (I-III). Alternative splicing of the C-terminal half of the third Ig-like domain generates the IIIb and IIIc isoforms in FGFRs1-3, but FGFR4 does not possess such alternative exons. There are reports of FGFRs 1–4 being expressed in CRC [6–11]. The FGF/FGFR family plays important roles in development and differentiation in normal human tissues, and the family is deeply involved in carcinogenesis and cancer progression in an autocrine or paracrine manner.

Recent studies have shown that gene amplification, abnormal activation, or single nucleotide polymorphisms (SNPs) of FGFR2 play important roles in cancer progression [12–16]; therefore, FGFR2 has been recognized as a novel therapeutic target for cancers [17]. The aim of this paper is to summarize the roles of FGFR2 and its isoforms in CRC and clarify whether they are potential therapeutic targets for CRC.

## 2. Structure of FGFR2 and Its Isoforms

An important feature and mode of regulation of FGFR2 functions is that structural variants of FGFR2 are generated by numerous alternative gene splicing events. These alternative splicing events of FGFR2 pre-mRNA generate mature mRNA, which encodes proteins altered in both the extracellular and intracellular regions. To date, more than 20 alternative splicing variants of FGFR2 have been identified [18].

**Figure 1:**
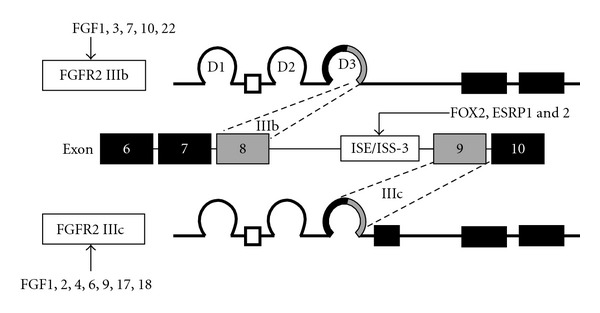
Alternative splicing of FGFR2 and specific ligands of each variant. The two FGFR2 isoforms are generated by alternative splicing of exons 8 and 9. This alternative splicing determines the specific ligands for each FGFR2 isoform [19]. FOX-2 or ESRP1 and 2 bind to the ISE/ISS-3 element to enhance splicing of exon 8 and silencing of exon 9.

The major splicing event occurs in the carboxyl terminal half of the third Ig-like domain (D3). The two types of FGFR2 isoforms are generated by alternative splicing of exons 8 and 9. When the C-terminal half of D3 is encoded by exon 8, the FGFR2 IIIb isoform is generated; while the FGFR2 IIIc isoform is generated when the C-terminal half of D3 is encoded by exon 9 (Figure 1) [19]. The Intronic Splicing Enhancer/Intronic Splicing Silencer-3 (ISE/ISS-3), which is located in intron 8 downstream of a UGCAUG motif of FGFR2, regulates the FGFR2 splicing [20] via binding of FOX-2 [21] or Epithelial Splicing Regulatory Protein (ESRP) 1 and 2 [22]. The ISE/ISS-3 functions specifically in epithelial cell types to enhance splicing of the upstream exon 8 and silencing of the downstream exon 9 [23]. Tissue-specific inclusion of either exon 8 or 9 generates either the epithelial cell-specific IIIb or mesenchymal cell-specific IIIc isoforms [24]. Subsequently, this alternative splicing determines the specific ligands for each FGFR2 isoform. FGF-1, 3, 7, 10, and 22 are reported to bind to FGFR2 IIIb; while FGF-1, 2, 4, 6, 9, 17, and 18 bind to FGFR2 IIIc with high affinity [19, 25, 26]. FGF-FGFR binding activates intracellular signaling cascades. Mitogenic signaling is mediated through tyrosine phosphorylation of key substrates, including activation of the mitogen-activated protein kinases, such as ERK-1 and ERK-2 via the ras pathway [27, 28].

The other major splicing of FGFR2 occurs in the sequence encoding the intracytoplasmic carboxyl terminus of FGFR2 IIIb. Three splice variants of FGFR2 IIIb have been identified. The variants are named C1, C2, and C3, and each has a different carboxyl-terminal sequence [29]. The C2-type carboxyl terminus is 34 amino acids shorter than the C1-type carboxyl terminus, and the C3-type carboxyl terminus is 19 amino acids shorter than the C2-type carboxyl terminus. These sequence differences result in distinct retention of tyrosine residues that may serve as sites for receptor autophosphorylation and docking sites for the cytoplasmic signaling proteins of FGFR2 IIIb. The FGFR2 IIIb-C3 isoform lacks the putative phospholipase C*γ* (PLC*γ*) binding site.

## 3. Expression and Roles of FGFR2 in Various Cancers

There are many published reports concerning the expression of FGFR2 in various cancers. Previous reports have shown overexpression of the C3 isoform in gastric cancer cell lines [29] and the C2 and C3 isoforms in breast cancer cell lines [30, 31], suggesting that aberrant expression of C2 or C3 splicing variants may contribute to cancer development.

Anomalous FGF signaling is associated with cancer development and progression. Gene amplification or missense mutations of FGFR2 occur in gastric, lung, breast, ovarian, and endometrial cancers and melanomas [8, 15, 18, 32–35]. SNPs of intron 2 in FGFR2 are associated with an increased risk of breast and endometrial cancers [12–14]. Furthermore, activating mutations of FGFR2 have been identified in approximately 10% of endometrial cancers, and inhibition of activated mutations of FGFR2 induced apoptosis and growth inhibition of endometrial carcinoma cells [15, 16]. In contrast, loss-of-function mutations of FGFR2 have been reported in melanomas [35]. These findings suggest that FGFR2 can play a context-dependent, opposing roles in various cancers.

A class switch from FGFR2 IIIb to FGFR2 IIIc is related to the progression of prostate cancers [36]. Furthermore, FGFR2 IIIc expression in prostate and bladder cancer cells induced epithelial-mesenchymal transition (EMT) and a switch in splicing, which may play crucial roles in cancer metastasis [37–39]. We previously reported that the expression levels of FGFR2 positively correlated with the presence of precancerous lesions in the uterine cervix, termed cervical intraepithelial neoplasia (CIN). Furthermore, stable transfection of FGFR2 IIIc in cervical cancer cell lines induced cancer cell growth [40]. Therefore, FGFR2 IIIc correlates with the carcinogenesis and aggressive growth of cervical cancer.

In contrast, the roles of the FGFR2 IIIb isoform have been controversial. Overexpression of the FGFR2 IIIb isoform, also known as keratinocyte growth factor receptor, has been reported in various cancers, including breast, endometrial, cervical, lung, esophageal, gastric, pancreatic, and CRC [10, 41–51]. We reported that expression of FGFR2 IIIb and one of its major ligands, FGF7, correlated with venous invasion, vascular endothelial growth factor A (VEGF-A) expression, and a poor prognosis and may promote venous invasion and tumor angiogenesis in pancreatic cancers [49]. Pancreatic cancer patients with high FGFR2 expression had a shorter survival time compared to those with low FGFR2 expression. Another ligand for FGFR2 IIIb, FGF10, induced pancreatic cancer cell migration and invasion via FGFR2 IIIb [52]. In contrast, decreased expression of FGFR2 IIIb in gastric cancer cells was associated with the proliferation and invasion of gastric cancer cells and a poor prognosis for the patient [47]. In esophageal cancers, FGFR2 IIIb expression correlated with a well-differentiated cell type, and FGF7 induced cell proliferation in FGFR2 IIIb positive cancer cells [10]. The different roles of FGFR2 IIIb in various cancers have not been well characterized; however, differences may be due to the affinity of different ligands for FGFR2 IIIb or effects from other FGFRs.

## 4. Expression and Roles of FGFR2 in CRC

**Figure 2:**
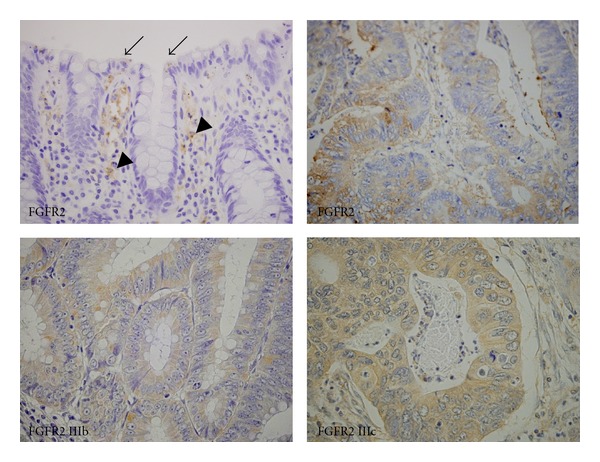
Immunohistochemical images of FGFR2 and its isoforms FGFR2 IIIb and FGFR2 IIIc in CRC. FGFR2 was expressed in the surface area of crypts in a nontumorous lesion obtained from CRC tissue (arrows). FGFR2 was also expressed in fibroblasts, inflammatory cells, and vascular endothelium (arrowheads). CRC cells exhibited marked expression of FGFR2 in the cell membrane and cytoplasm. Isoforms of FGFR2, FGFR2 IIIb, and FGFR2 IIIc were expressed in CRC cells. Original magnification: upper left panel, ×200; other panels, ×600.

Immunohistochemical analysis has demonstrated that FGFR2 is expressed in the differentiated cells located on the upper portion of the intestinal crypt in normal colorectal epithelium (Figure 2, arrows) [53] and is related to the proliferation and differentiation of cells [10, 54]. In patients with CRC, expression of FGFR2 protein was observed in the cell membrane and cytoplasm of cancer cells (Figure 2). The mRNA of several FGFs, including FGF2, 8, 9, and 18, was expressed in CRC cell lines and was detected by RT-PCR analysis (Table 1) [49]; therefore, FGFs/FGFRs may regulate CRC cell growth in an autocrine manner. Recently, we reported that the invasive front of CRC cells exhibited stronger FGFR2 expression than the surface or central area of the cancerous cells [53]. Decreased expression of FGFR2 in CRC cells was associated with inhibited cell migration, invasion, and tumor growth *in vitro* and *in vivo *[53]. Thus, FGFR2 plays important roles in CRC progression in correlation with tumor cell migration, invasion, and growth (Table 2).

Overexpression of the FGFR2 isoform FGFR2 IIIb in CRC was associated with a well-differentiated histological type [10, 55]. As determined by* in situ *hybridization, both FGFR2 IIIb and FGF7 mRNA are expressed in CRC cells, and FGF7 mRNA was recognized in neuroendocrine cells lying close to CRC cells [50]. FGF7 induced an increase of VEGF-A; thus, FGF-7 and its receptor FGFR2 IIIb may be involved in tumor angiogenesis in CRC [56]. Furthermore, FGF7 enhances adhesion to type-IV collagen, one of the main components of the vascular basement membrane, via downregulation of Integrin *α*2 and activation of ERK1/2 and focal adhesion kinase (FAK) signaling pathways [57]. Coexpression of FGFR2 IIIb and FGF10 has been reported in CRC cells, and FGF10 increased the growth rate of FGFR2 IIIb-positive CRC cell lines [51]. These findings suggest interactions of FGFR2 IIIb, FGF7, and FGF10 may play important roles in CRC proliferation and tumor angiogenesis via autocrine and/or paracrine manners (Table 2).

**Figure 3:**
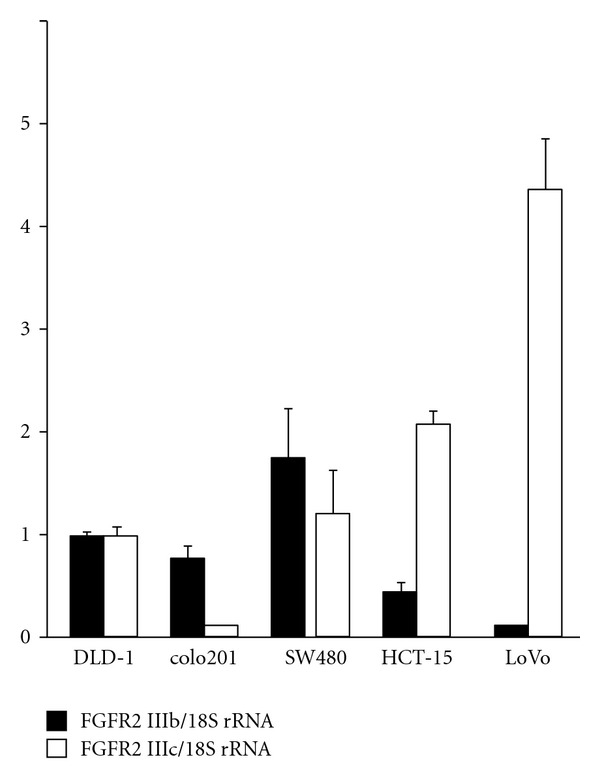
Expression levels of FGFR2 IIIb and IIIc in CRC cell lines. All 5 CRC cell lines expressed FGFR2 IIIb and IIIc mRNAs at various levels. Quantitative RT-PCR results were expressed as the ratio of target to 18S rRNA, with the latter serving as an internal standard. Gene expression levels were measured in triplicate.

**Figure 4:**
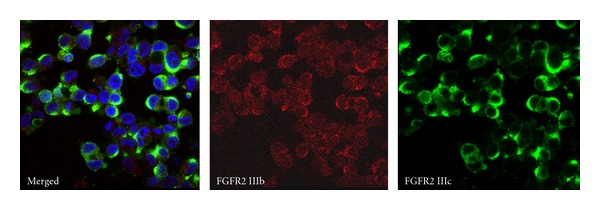
Immunocytochemical analysis of FGFR2 IIIb and IIIc in CRC cell lines. A human CRC cell line, LoVo, exhibited both FGFR2 IIIb and FGFR2 IIIc isoforms in the cell membrane and cytoplasm. The expression level of FGFR2 IIIb was weak, but IIIc was strong, which is consistent with their mRNA levels. Original magnification, ×1000; red, FGFR2 IIIb; green, FGFR2 IIIc; blue, DAPI.

Immunohistochemical analysis, quantitative RT-PCR, and fluorescent staining determined that both FGFR2 IIIb and FGFR2 IIIc are expressed in CRC tissues and cell lines (Figures 2, 3, and 4) [49, 58]. However, the roles of the FGFR2 IIIc isoform in CRC are not clear and require further examination. 

## 5. Molecular-Targeted Therapy of the FGFR2 Signaling Pathway

Several kinds of molecular-targeted therapies to the FGFR2 signaling pathway have been reported in various cancers. In endometrial cancer cells harboring activated mutations of FGFR2, knockdown of FGFR2 using short hairpin (sh) RNA or treatment with a pan-FGFR inhibitor, PD173074, caused cell cycle arrest and cell death [15]. A small molecule, Ki23057, which inhibits autophosphorylation of FGFR2 IIIb decreased the growth of biliary tract cancer cells [59], gastric scirrhous carcinoma cells [60, 61], and CRC cells [62] *in vitro* and *in vivo*. A recent study demonstrated that monoclonal antibodies to FGFR2 IIIb and FGFR2 IIIc isoforms successfully inhibited the growth of gastric tumor xenografts [63]. A mutation in the soluble ectodomain of FGFR2 IIIc, S252W, suppressed cell growth, angiogenesis, and metastasis of human breast cancer and prostate cancer cell lines *in vitro* and *in vivo* [64]. We recently reported that shRNA-targeting FGFR2 in CRC cell lines suppressed cancer cell growth, migration, and invasion [53]; however, no other reports have shown the usefulness of FGFR2-targeting therapy in CRC. The best way for cancer treatment to inhibit FGFR2 IIIb or FGFR2 IIIc, or both isoforms, has not been determined. It may depend on each cell line or individuals that express different endogenous FGFR2 levels, and other FGFRs and FGFs. Basic research clearly indicates the effectiveness of FGFR2 as a target for CRC therapy; therefore, clinical studies are needed to develop a novel therapeutic strategy in CRC.

## 6. Other FGFRs and FGFs in CRC

All four FGFRs are reportedly expressed in CRC [6–11]. FGFR1 and FGFR2 are expressed in both colorectal adenomas and CRC, and expression might indicate the transformation from human colon adenoma to carcinoma [6]. Overexpression of the FGFR1 gene leads to liver metastasis in CRC [9].

FGF2 and FGF20 are implicated in embryogenesis and tissue regeneration within the colon [65–67]. FGF2 is expressed in submucosal tissues to induce angiogenesis, while FGF20 is expressed in epithelial progenitor cells to induce epithelial proliferation. Aberrant splicing and activation of cryptic splice sequences in FGFR3 have been identified in patients with CRC [8, 68]. FGFR4 and its specific ligand FGF19 are coexpressed in cancer cells and induce growth via ERK and FRS2 in CRC and hepatocellular carcinoma [69]. In human intestinal epithelial cell lines and CRC cell lines, a soluble splice variant of FGFR4 has been reported [70], but its roles have not been determined.

## 7. Molecular-Targeted Therapy to Other FGF/FGFR Signaling Pathways

Protein kinases all have a catalytic domain with a conserved amino acid sequence and similar three-dimensional structures [71]. Protein kinases are now the most popular class of drug target after G protein-coupled receptors [72]. Small-molecule compounds with multiple targets have recently been developed to overcome the recurrence of drug-resistant tumors [73]. There are several inhibitors of FGFR1 tyrosine kinase activity, including PD173074, SU5402, and AZD2171. AZD2171 inhibits VEGFR2, PDGFR*β*, and FGFR1 tyrosine kinase activity, while PD173074 and SU5402 target a relatively narrow range of tyrosine kinases. In a mouse-xenografted CRC tumor, combined therapy with a recombinant FGFR1 protein vaccine and low-dose gemcitabine inhibited tumor growth and antiangiogenesis was present [74]. TSU68, a multiple-receptor tyrosine kinase inhibitor that targets FGFRs, vascular endothelial growth factor receptor-2 (VEGFR2), and a platelet-derived growth factor receptor (PDGFR) inhibited CRC cell growth via normalization of tumor vessels [75]. Brivanib Alaninate is an orally available, selective tyrosine kinase inhibitor that targets both VEGFR-2 and FGFR1 [76]. ENMD-2076, a small-molecule kinase inhibitor with activity against the Aurora kinases A and B and several other tyrosine kinases, including VEGFR-2, cKit, and FGFR1, inhibited tumor growth in murine xenograft models of human CRC [77].

An anti-FGFR3 monoclonal antibody that inhibited not only wild-type FGFR3, but also various mutants of the receptor, exerted potent antitumor activity against bladder carcinoma and multiple myeloma xenografts in mice [78]. The human single-chain variable fragment (Fv) directed against FGFR3 blocked the proliferation of bladder carcinoma cells *in vitro* and *in vivo *[79]. Currently, most small molecule compounds inhibit multiple tyrosine kinases and may affect not only cancer cells but also normal cells. To develop more selective tyrosine kinase inhibitors, monoclonal antibodies, vaccines, and emerging technologies, such as RNA aptamers or siRNA, will be needed to treat advanced CRC.

## 8. Conclusion

FGFR2 and its isoform are highly expressed in CRC and correlate with CRC growth, invasion, and tumor angiogenesis. The effectiveness of FGFR2-targeting therapy for CRC has been clearly demonstrated *in vitro* and *in vivo* in basic research studies, more so than for other FGFs or FGFRs. Thus, anti-FGFR2 therapies, including selective targeted therapy to the isoforms, may be novel treatments for advanced CRC patients.

## Figures and Tables

**Table 1:** Expression of FGFs in CRC cell lines.

	FGF1	FGF2	FGF4	FGF6	FGF8	FGF9	FGF17	FGF18
DLD-1	−	+	−	−	+	−	−	+
HCT-15	−	+	−	−	+	+	−	+
SW480	−	−	−	−	+	+	−	+
LoVo	−	+	−	−	+	+	−	−
Colo201	−	−	−	−	+	+	−	+

**Table 2:** Roles of FGFR2 and its splicing variants in colorectal cancers.

		Reported data	Roles
FGFR2	normal	located in the upper portion of the intestinal crypt [50, 54]	differentiation, growth
	cancer	increased expression in the invasive front of cancer cells	invasion, growth
		shRNA-targeting FGFR2 suppressed cell growth [53]	

FGFR2 IIIb	normal	located in the upper portion of the intestinal crypt [50]	differentiation, growth
	cancer	Well-differentiated types [50]	differentiation
	cancer	FGF-7 induced an increase of VEGF-A [56]	angiogenesis
	cancer	FGF7 enhanced the adhesion to type-IV collagen [57]	cell attachment
	cancer	FGF10 increased the growth rate [51]	growth

FGFR2 IIIc	cancer	expressed in cancer cells (the present report)	not reported
